# Some interface theories and Hall–Petch relationship

**DOI:** 10.1098/rsos.241954

**Published:** 2025-05-14

**Authors:** Hiroaki Yokoyama, Hiroyuki Nagahama

**Affiliations:** ^1^ Department of Earth Science, Tohoku University, Sendai, Japan

**Keywords:** Hall–Petch relationship, deformation twin, dislocation density, stress, surface dislocation, rank-1 connection

## Abstract

In polycrystalline materials, an increase in the number of interfaces strengthens the material. For example, in deformation twins, deformation experiments have shown a correlation between the deformation-twin density and flow stress. However, this relationship is empirical, and its theoretical background is not yet well understood. In this study, we explain the relationship between interface density (particularly deformation-twin density) and flow stress using interface theories. From macroscopic and mathematical perspectives, the density of the surface dislocation is equivalent to the dislocation density with differential dimensions. Therefore, the relationship between the deformation-twin density and the flow stress obtained from deformation experiments on calcite aggregates can be derived from the correspondence between the dislocation density and the deformation-twin density. Additionally, we demonstrate the mathematical equivalence between surface dislocation theory and other interface theories (rank-1 connection, Hadamard jump condition or 0-lattice theory) as applied to deformation-twin, martensite, kink and grain boundaries. Furthermore, the Hall–Petch relationship and inverse Hall–Petch relationship can be explained from the perspective of the rank-1 connection.

## 1. Introduction

The formation of deformation twins affects the mechanical properties of materials, such as metals and minerals, by increasing the interface and affecting dislocation movement [1–5]. For example, in calcite, deformation experiments have reported a relationship between the linear density of deformation twins (hereinafter referred to as deformation-twin density) and flow stress, which is expressed as


(1.1) $$\begin{array}{lcr} & \sigma =\bar{C}\sqrt{\rho }\text{,} & \end{array}$$

where σ represents the flow stress, ρ represents the deformation-twin density (number of deformation twins/length [mm]) and $\bar{C}$ is a constant [6]. Equation (1.1) is equivalent to the Bailey–Hirsch equation [7] (also referred to as the Taylor equation [8]) and is used in palaeostress estimation for geologically deformed calcite [9–12]. Similar relationships between deformation-twin density and flow stress are also reported in metals and alloys [13,14]. A comparable relational expression has also been recognized between the geometric mean grain size and flow stress in plastically deformed polycrystals as follows:


(1.2) $$\sigma =\sigma_{0}+\frac{k}{\sqrt{d}},$$

where σ is the flow stress; d is the geometric mean grain size; $\sigma_{0}$ and k are constants related to the materials, which are the internal stress and resistance of the interface to slip propagation (Hall–Petch coefficient), respectively. Equation (1.2) is known as the Hall–Petch relationship [15,16]. The Hall–Petch relationship is important when considering strain hardening, as the strength increases as the grain size decreases [17,18]. However, the Hall–Petch relationship does not hold for nanometric grain sizes, and it has been reported that the smaller the grain size, the smaller the yield stress [18–20]. This is known as the inverse Hall–Petch relationship, and the mechanism has been extensively discussed in materials science [18–20]. However, the Hall–Petch relationship and inverse Hall–Petch relationship are both empirical equations, and their theoretical or mathematical backgrounds are not well understood yet.

The key to solving this problem lies in the interfaces (deformation-twin boundary or grain boundary) and their connection conditions. Surface dislocation theory is one of the interface theories, originally developed for martensite interfaces [21]. This theory states that two different crystal lattices are microscopically connected by surface dislocations [21]. The deformation-twin boundary is the interface between the matrix and deformation twin, which is formed by lattice rotation (i.e. $52^{\circ }$ for calcite [22]) when the crystal is sheared [22,23]. Although they are different in crystallography, martensite, deformation twins and kinks are formed from crystallographic properties and are geometrically equivalent. The surface dislocation theory can be applied to the interfaces formed by these, and it is known that they cause strain hardening [24–28]. In addition, rank-1 connection [29], Hadamard jump condition [30] and 0-lattice theory [31] are also referred to as interface theories that describe the state of interfaces.

In this study, we aim to provide a theoretical understanding of hardening and softening behaviour due to interfaces such as deformation-twin boundaries and grain boundaries. In §2, we review surface dislocation theory and discuss its application to deformation twins. In §3, we introduce the geometrical conditions of compatibility of the first order for discontinuity and their mathematical equivalence to the Hadamard jump conditions, which are consistent with the surface dislocation theory. In §4, we introduce the relationship between the flow stress and dislocation density, which can be expressed using tensor notation and can be applied to the relationship between the flow stress and deformation-twin density in tensor notation. Finally, the physical background of the relationship between flow stress and deformation-twin density and the relationship between grain size and flow stress are shown. This is consistent with the experimental results for deformation-twin density and flow stress of calcite aggregates. In addition, the hardening and softening behaviour due to deformation-twin, martensite, kink and grain boundaries are explained from the perspective of the rank-1 connection. Furthermore, we show the equivalence among rank-1 connection, surface dislocation theory, geometrical conditions of compatibility of the first order for discontinuity, Hadamard jump condition and 0-lattice theory.

In the tensor notation used in this article, we distinguish between covariant and contravariant indices, but there is no need to make this distinction when thinking in an orthogonal linear coordinate system. Also, regarding the subscripts, Einstein’s summation convention is used.

## 2. Surface dislocation theory for martensite and deformation-twin boundaries

Bullough & Bilby [21] proposed the surface dislocation theory for the analysis of martensite crystallography. For its application, the surface dislocation that defines the boundary between phases is assumed to correspond to a parallel array of identical dislocations with an arbitrary Burgers vector moving in an arbitrary plane, with the Burgers vector and plane being assumed as given [21]. This theory is briefly reviewed below according to the notations of Bullough & Bilby [21], and is applied to the deformation twin.

Let ${\hat{x}}_{1}$, ${\hat{x}}_{2}$ and ${\hat{x}}_{3}$ be a set of orthogonal unit vectors defining the orthogonal Cartesian coordinate system, and let $x=x^{i}{\hat{x}}_{i}$ be any vector. Consider a surface whose unit normal is $\nu =\nu^{i}{\hat{x}}_{i}$. Two lattices are generated on the positive and negative sides of this normal ν with their bases labelled $a^{(+)i}$ and $a^{(-)i}$, respectively. These lattices are created through uniform deformation of a third reference lattice. Let $D_{j}^{(+)i}$ and $D_{j}^{(-)i}$ represent the deformations in the Cartesian system ${\hat{x}}_{i}$; then, any vector $x=x^{i}{\hat{x}}_{i}$ can be divided into the two regions: $x^{\left(+\right)}$ and $x^{\left(-\right)}$. The dislocation content of the plane boundary between the lattices is then expressed as


(2.1) $$B_{j}^{i}=\varepsilon_{jk}^{l}\nu^{k}\left[E_{l}^{(+)i}-E_{l}^{(-)i}\right]\text{,}$$

where $B_{j}^{i}{\hat{x}}_{i}$ is the resultant Burgers vector of the dislocation lines that cut the unit length of a line within the boundary perpendicular to the $x=x^{j}$ direction. Here, $\varepsilon_{jk}^{l}$, which is the Levi‐Civita tensor, vanishes unless j, k and l are all different and have a value of +1 or −1, depending on whether j, k and l are even or odd permutations of the numbers 1, 2 and 3. Matrices $E_{j}^{(+)i}$ and $E_{j}^{(-)i}$ are reciprocals of $D_{j}^{(+)i}$ and $D_{j}^{(-)i}$, respectively.

Let p be a unit vector on a boundary surface. Then, the component of the unit vector w normal to p within the boundary is $w^{i}=\varepsilon_{kl}^{i}\nu^{k}p^{l}$, and the resulting Burgers vector dislocation line cut at p is $B_{j}^{i}w^{j}\hat{x}$. The relationship between the Burgers vector and deformation gradient, when the martensite matrix is not deformed, can be expressed as


(2.2) $$\begin{array}{rl} B^{i} & =B_{j}^{i}w^{j}\\ & =\varepsilon_{jk}^{l}\nu^{k}\left[E_{l}^{(+)i}-E_{l}^{(-)i}\right]\varepsilon_{mn}^{j}\nu^{m}p^{n}\\ & =\left(E_{l}^{(+)i}-\delta_{l}^{i}\right)p^{l}\text{,} \end{array}$$

where $\varepsilon_{jkl}\varepsilon_{jmn}=\delta_{km}\delta_{ln}-\delta_{kn}\delta_{lm}$ and $\nu^{n}p^{n}=0$.

Bullough & Billby [21] stated that crystals with two different lattice orientations are bound by surface dislocations. Deformation-twin boundaries are similarly bounded by surface dislocations [32], and this concept has been applied to deformation twins. Although Bullough & Billby [21] expressed $B_{j}^{i}$ as the dislocation content, $B_{j}^{i}$ is similar to the microscopic dislocation density on the interface. Furthermore, these surface dislocations should be oriented in various directions within an object. Hence, macroscopically, surface dislocations are used as normal dislocations because of the following reasons.

In the continuum theory of defects [33,34], a Burgers circuit of unit area normal to the unit vector $n^{j}$ has a Burgers vector $B^{i}$, and $B^{i}$ is expressed as follows [35]:


(2.3) $$\begin{array}{lcr} & B^{i}=\alpha_{j}^{i}n^{j}\text{,}\;\alpha_{j}^{i}=-\varepsilon_{kl}^{i}\left(\frac{\partial \beta_{j}^{l}}{\partial x_{k}}\right)\text{,} & \end{array}$$

where $\alpha_{j}^{i}$ denotes the dislocation density tensor (Nye tensor) and $\beta_{j}^{l}$ is plastic distortion (plastic part of displacement gradient). Moreover, the total Burgers vectors $B_{\text{total}}^{i}$ of the surface dislocations in an arbitrary area s are given as follows [35–38]:


(2.4) $$B_{\text{total}}^{i}=\int_{s}\alpha_{j}^{i}n^{j}\text{d}s.$$

Many dislocations are deposited in the dislocation content as surface dislocations in the deformation-twin boundaries (i.e. $B_{j}^{i}$ in equation (2.1)). Comparing equations (2.2) and (2.3), the continuum dislocation density tensor $\alpha_{j}^{i}$ and surface dislocation density tensor $B_{j}^{i}$ are dimensionally different, but the dislocation density tensor $B_{j}^{i}$ for surface dislocations can be treated as the dislocation density tensor $\alpha_{j}^{i}$ for the deformation-twin densities of metals and minerals.

## 3. Geometrical conditions of compatibility of the first order for discontinuity

Using the description [39,40], the following equation is derived based on Thomas [40] and Yokomichi [41,42]. Consider a moving surface R in continuum defined by the Cartesian coordinates $x^{i}$ (i is 1, 2 and 3), defined by curvilinear coordinates of the surface $u^{\overset{ˇ}{a}}$ ($\overset{ˇ}{a}$ is 1, 2) and time t as follows:


(3.1) $$\begin{array}{lcr} & x^{i}=x^{i}\left(u^{1},u^{2},t\right). & \end{array}$$

It is assumed that the function $x^{i}$ is continuous and differentiable in $u^{\overset{ˇ}{a}}$, and that the surface R is regular with the function matrix ($\partial x^{i}/\partial u^{\overset{ˇ}{a}}$) always having rank two; this condition is sufficient for the existence of a tangent plane at each point of R(t) and for the local representation of the surface, at any time t, by means of an equation involving only the x coordinates. The differential coefficients owing to $u^{\overset{ˇ}{a}}$ are also expressed as follows:


(3.2) $$\begin{array}{lcr} & x_{\overset{ˇ}{a}}^{i}=\frac{\partial x^{i}}{\partial u^{\overset{ˇ}{a}}}\text{,}\;x_{\overset{ˇ}{a}\overset{ˇ}{b}}^{i}=\frac{\partial^{2}x^{i}}{\partial u^{\overset{ˇ}{a}}\partial u^{\overset{ˇ}{b}}}\text{,} & \end{array}$$

where $x_{\overset{ˇ}{a}}^{i}$ represents the partial derivative of $x^{i}$ with respect to $u^{\overset{ˇ}{a}}$. Let Φ be a physical quantity defined as a function of the coordinate $x^{i}$ in the continuum. Here, Φ is a discontinuity expressed as $[\Phi ]=\Phi_{2}-\Phi_{1}$ when the values on two sides of the surface R are $\Phi_{1}$ and $\Phi_{2}$. Additionally, $\left[\Phi_{;i}\right]$ is defined as the first-order geometric condition for discontinuity Φ, and $\left[\Phi_{;i}\right]$ represents the covariant derivative of Φ with $x_{i}$. Moreover, $\left[\Phi_{;i}\right]$ is defined as the first-order geometric condition for discontinuity when $[\Phi ]=\Phi_{2}-\Phi_{1}$, and its values on the two sides of the surface R are $\Phi_{1}$ and $\Phi_{2}$. For simplicity, we assume the following:


(3.3) $$\begin{array}{lcr} & H=[\Phi ]\text{,}\;T=[\Phi_{;i}]{\hat{\nu }}^{i}\text{,} & \end{array}$$

where ${\hat{\nu }}^{i}$ denotes the unit normal vector of the surface R.

From the above definitions, considering the necessary assumptions regarding the existence of the derivatives of Φ in spatial coordinates $x^{i}$ and the curvilinear coordinates of the surface $u^{\overset{ˇ}{a}}$ [43], we obtain


(3.4) $$H_{;\overset{ˇ}{a}}=[\Phi_{;\overset{ˇ}{a}}]=[\Phi_{;i}]x_{\overset{ˇ}{a}}^{i}.$$

The following relationship exists regarding the metric tensor $g^{\overset{ˇ}{a}\overset{ˇ}{b}}$ on a surface R [43]:


(3.5) $$\begin{array}{lcr} & g^{\overset{ˇ}{a}\overset{ˇ}{b}}x_{\overset{ˇ}{a}}^{i}x_{\overset{ˇ}{b}}^{j}=\delta^{ij}-{\hat{\nu }}^{i}{\hat{\nu }}^{j}\text{,} & \end{array}$$

where $\delta^{ij}\left(\equiv g^{im}g_{m}^{j}\right)$ denotes the Kronecker delta. Here, by multiplying the left and right members of equation (3.4) by $g^{\overset{ˇ}{a}\overset{ˇ}{b}}x_{\overset{ˇ}{b}}^{i}$ and applying equation (3.5), we obtain


(3.6) $$[\Phi_{;i}](\delta^{ij}-{\hat{\nu }}^{i}{\hat{\nu }}^{j})=g^{\overset{ˇ}{a}\overset{ˇ}{b}}H_{;\overset{ˇ}{a}}x_{\overset{ˇ}{b}}^{j}.$$

Therefore, considering that $\delta^{ij}=0$ except when i=j, and introducing the symbol T defined by equation (3.3), we have


(3.7) $$\begin{array}{lcr} & \left[\Phi_{;i}\right]=T{\hat{\nu }}_{i}+g^{\overset{ˇ}{a}\overset{ˇ}{b}}H_{;\overset{ˇ}{a}}x_{i\overset{ˇ}{b}}. & \end{array}$$

Equation (3.7) is called the *geometrical conditions of compatibility of the first order* for discontinuity H [40]. In case H is continuous across surface R, then H=[Φ]=0 from equation (3.3), hence equation (3.7) becomes


(3.8) $$\begin{array}{lcr} & \left[\Phi_{;i}\right]=T{\hat{\nu }}_{i}. & \end{array}$$

In equation (3.8), although T is a scalar function, if we replace T with a vector function $T_{i}$, we obtain


(3.9) $$\begin{array}{lcr} & \left[\Phi_{i;j}\right]=T_{i}{\hat{\nu }}_{j}. & \end{array}$$

When equation (3.9) is a jump function for the deformation gradient with respect to the surface R, and is equivalent to the Hadamard jump condition (equation (A 2)). In particular, if Φ is continuous across the surface R, the geometrical conditions of compatibility of the first order for the discontinuity are attributed to the Hadamard jump condition. Moreover, by comparing equations (2.1) and (3.9), equation (3.9) becomes mathematically equivalent to equation (2.1).

## 4. Flow stress and dislocation density relation in tensor notation

The effects of increasing dislocation density on the mechanical properties of materials have been studied for many years [8,44], and the Bailey–Hirsch type relationship between dislocation density and flow stress is expressed as follows:


(4.1) $$\begin{array}{lcr} & \sigma =\tilde{A}\mu B\sqrt{\alpha }\text{,} & \end{array}$$

where σ represents the flow stress, μ represents the shear modulus, B represents the magnitude of the Burgers vector, $\tilde{A}$ is a constant and α represents the scalar dislocation density [7]. Here, dislocations are originally classified as edge dislocations or screw dislocations. However, the scalar dislocation density α in equation (4.1) does not contain information about the dislocation types because it represents the statistically stored dislocation density. Therefore, it does not represent geometric properties such as geometrically necessary dislocations (GN dislocations), and the effect of stress on the dislocation type cannot be determined from equation (4.1).

In order to solve the above problem, Yamasaki [45] derived equation (4.1) in tensor notation based on the following assumptions: (i) the higher-order terms and spatial derivatives of dislocation density can be ignored and (ii) the material is isotropic. Here, we briefly introduce the derivation of the tensor notation for equation (4.1), according to the notation of Yamasaki [45]. In the Euclidean material space where defects do not exist, we use the coordinate system {$x^{i}$}. In the non-Euclidean material space where a defect exists, we use the coordinate system {$q^{\mu }$}. Let $\Delta x^{i}$ be the difference between {$x^{i}$} and {$q^{\mu }$}: $q^{i}=x^{i}+\Delta x^{i}$. The displacement tensor is related to the stress tensor $\sigma^{\chi \gamma }$ using the generalized Hooke’s law $\sigma^{\chi \gamma }(x)=C^{\chi \gamma \xi \mu }E_{\xi \mu }(x)$, where $C^{\chi \gamma \xi \mu }$ is the elastic coefficient and $E_{\xi \mu }$ represents the displacement gradient. Therefore, the relationship between flow stress and dislocation density is derived as follows:


(4.2) $$\begin{array}{lcr} & \sigma^{\chi \gamma }(x)=C^{\chi \gamma \xi \mu }\left[E_{\xi \mu }^{\prime }(q)+\frac{1}{2}e_{\xi \kappa }\varepsilon_{\eta v\mu }\alpha^{\eta \kappa }\Delta x^{v}\right]\text{,} & \end{array}$$

where the displacement gradient $E_{\xi \mu }^{\prime }(q)=\delta_{\xi \mu }-F_{\xi \mu }^{\prime }(q)$, and $e_{\mu }^{i}(q)=F_{\mu }^{\prime i}(q)=\partial x^{i}/\partial q^{\mu }$. Here, $F_{\xi \mu }^{\prime }(q)$ denotes the deformation gradient tensor affected by the defect field. Moreover, we divide $\sigma^{\chi \gamma }$, $E^{\prime \chi \gamma }$ and $\alpha^{\eta \chi }$ into the mean, hydrostatic or deviatoric components:


(4.3) $$\begin{array}{lcr} & \sigma^{\chi \gamma }={\tilde{\sigma }}^{\chi \gamma }+\delta^{\chi \gamma }\sigma^{\prime }/3\text{,}\;E^{\prime \chi \gamma }={\tilde{E}}^{\prime \chi \gamma }+\delta^{\chi \gamma }E^{\prime \prime }/3\text{,}\;\alpha^{\eta \chi }={\tilde{\alpha }}^{\eta \chi }+\delta^{\eta \chi }\alpha^{\prime }/3\text{,} & \end{array}$$

where $\sigma^{\prime }\equiv \sigma_{\chi }^{\chi }$, $E^{\prime \prime }\equiv E_{\mu }^{\prime \mu }$, and $\alpha^{\prime }\equiv \alpha_{\eta }^{\eta }$. In this case, we obtain the following from equation (4.2):


(4.4) $$\begin{array}{lcr} & \begin{array}{rl} {\tilde{\sigma }}^{\chi \gamma }(x) & =2\mu {\tilde{E}}^{\prime (\chi \gamma )}(q)+\mu e^{\left(\chi \right|}{,}_{\kappa }\varepsilon_{\eta v}{,}^{\left|\gamma \right)}{\tilde{\alpha }}^{\eta \kappa }\Delta x^{v}\\ & =2\mu {\tilde{E}}^{\prime (\chi \gamma )}(q)+\frac{1}{3}\mu e^{(\chi |\eta }\varepsilon_{\eta v}{,}^{\left|\gamma \right)}\alpha^{\prime }\Delta x^{v}+\mu e^{\left(\chi \right|}{,}_{\kappa }\varepsilon_{\eta v}{,}^{\left|\gamma \right)}{\tilde{\alpha }}^{\eta \kappa }\Delta x^{v}\text{,} \end{array} & \end{array}$$

where $E^{\prime (\chi \gamma )}\equiv \left(E^{\prime \chi \gamma }+E^{\prime \gamma \chi }\right)/2$, $e^{\left(\chi \right|}{,}_{\kappa }\varepsilon_{\eta v}{,}^{\left|\gamma \right)}\equiv \left(e_{\kappa }^{\chi }\varepsilon_{\eta v}^{\gamma }+e_{\kappa }^{\gamma }\varepsilon_{\eta v}^{\chi }\right)/2$, κ=λ+(2/3)μ and μ and λ are Lame’s constants (Lame’s constant μ is different from the superscript μ of the coordinate system {$q^{\mu }$}). From equation (4.4) and a Taylor series, i.e. ${\tilde{\sigma }}^{\chi \gamma }(x)={\tilde{\sigma }}^{\chi \gamma }(q-\Delta x)={\tilde{\sigma }}^{\chi \gamma }(q)-\partial_{v}{\tilde{\sigma }}^{\chi \gamma }\Delta x^{v}+\cdots$, we have


(4.5) $$\begin{array}{lcr} & \partial_{v}{\tilde{\sigma }}^{\chi \gamma }=-\mu e^{\left(\chi \right|}{,}_{\kappa }\varepsilon_{\eta v}{,}^{\left|\gamma \right)}{\tilde{\alpha }}^{\eta \kappa }. & \end{array}$$

Czechowski *et al*. [46] used two equations: $\partial_{x}\sigma =f_{1}\left(\sigma ,\sigma_{R},V\right)$ and $V=f_{2}\left(\sigma ,\sigma_{R}\right)$*,* where $f_{1}\left(\sigma ,\sigma_{R},V\right)$ and $f_{2}\left(\sigma ,\sigma_{R}\right)$ are the polynomial expressions for flow stress σ, resistance stress $\sigma_{R}$ and dislocation velocity V. From these two equations, $\partial_{x}\sigma =f_{1}\left(\sigma ,\sigma_{R},f_{2}\left(\sigma ,\sigma_{R}\right)\right)=f_{3}\left(\sigma ,\sigma_{R}\right)$. Czechowski *et al*. [46] showed that $f_{3}\left(\sigma ,\sigma_{R}\right)\propto \sigma^{2}$ in the particular case of $\sigma \gg \sigma_{R}$. Hence


(4.6) $$\begin{array}{lcr} & {\tilde{\sigma }}_{\rho v}{\tilde{\sigma }}^{\rho \gamma }=\mu A_{\chi }^{\prime }e^{\left(\chi \right|}{,}_{\kappa }\varepsilon_{\eta v}{,}^{\left|\gamma \right)}{\tilde{\alpha }}^{\eta \kappa }\text{,} & \end{array}$$

where ${\tilde{\sigma }}_{\rho v}{\tilde{\sigma }}^{\rho \gamma }$ is the tensor notation of $\sigma^{2}$ and ${\tilde{\alpha }}^{\eta \kappa }$ denotes the dislocation density tensor. Therefore, equation (4.6) is the tensor notation for equation (4.1). If ${\tilde{\sigma }}^{\chi \gamma }x_{\gamma }$ is proportional to the strain energy of dislocation $\mu B_{i}B^{i}$, then $A_{\chi }^{\prime }={\tilde{\sigma }}_{\chi \gamma }x^{\gamma }=\mu B_{i}B^{i}A_{\chi }$. The right side of equation (4.6) indicates the type of dislocation; (η≠χ) represents edge dislocations and (η=χ) represents screw dislocations [45].

## 5. Discussion

In this section, the physical background of equation (1.1) is discussed in comparison with the relationship between flow stress and dislocation density. As indicated by equations (4.1) and (4.6), the flow stress is proportional to the square root of the dislocation density. In the continuum theory of defects, the dislocation density tensor ${\tilde{\alpha }}^{\kappa \lambda }$, which corresponds to the antisymmetric part of the connection $\Gamma_{\left[\mu v\right]}^{\lambda }\equiv \left(\Gamma_{\mu v}^{\lambda }-\Gamma_{v\mu }^{\lambda }\right)/2$, is expressed as follows [33,34,45]:


(5.1) $${\tilde{\alpha }}^{\kappa \lambda }=\varepsilon^{\kappa \mu v}\Gamma_{\left[\mu v\right]}^{\lambda }=\varepsilon^{\kappa \mu v}S_{\mu v}^{\lambda }$$

where $S_{\mu v}^{\lambda }=\Gamma_{\left[\mu v\right]}^{\lambda }$ denotes the torsion tensor. Therefore, the dislocation density tensor in equation (4.6) can be expressed in terms of the torsion tensor. From the viewpoints of non-Riemann space [36–38], equations (2.3), (2.4) and (5.1) indicate that the dislocation or the Burgers vector can be represented by the torsion tensor as the topological singularity due to the multi-valued displacement vector. The torsion is equivalent to the Clairaut–Schwarz–Young’s integrability condition. Moreover, as mentioned in Scheidegger [47], a derivative of displacement vector is related to the Cauchy–Riemann equation [48,49]. Then, a path dependency of the displacement can be given by the torsion tensor. In this case, the topological singularities such as the Burgers vector are path-dependent, and the domain is not simply connected from Poincaré’s lemma or the de Rham cohomology. This geometric approach is applicable to a finite deformation in non-classical elasticity. Instead of spatial coordinates, the geometric objects expressed by the material coordinates give non-integrability conditions. Then, the singularity due to the non-integrability of multi-valued fields can be studied as the boundary value problem in the non-Riemann space (see [37] for details).

Shiozawa & Ohnami [50] experimentally investigated the relationship between resolved shear stress and dislocation density using the continuum theory of defects [33,34] and the X-ray back-reflection Laue method. The resolved shear stress τ was calculated using the Schmid factor by calibration with the tensile strain $\varepsilon_{T}$ as follows:


(5.2) $$\begin{array}{lcr} & \tau =L\frac{\cos\;\phi }{s_{c}}\frac{\sqrt{{\left(1+\varepsilon_{T}\right)}^{2}-\sin^{2}\;\omega }}{1+\varepsilon_{T}}\text{,} & \end{array}$$

where L represents the axial load, $s_{c}$ represents the cross-sectional area of the test specimen before deformation, ϕ represents the angle between the direction of tension and the normal direction of the slip plane and ω represents the angle between the direction of tension and the slip direction [50]. Although equation (4.6) is expressed as the flow stress, this relationship also holds for the resolved shear stress [50]. Therefore, from equations (4.6), (5.1) and (5.2), the resolved shear stress is expressed by the torsion tensor $S_{12}^{i}(i=1,2,3)$ as follows:


(5.3) $$\tau^{31}=1.63\times 10^{-4}\sqrt{S_{12}^{1}}+1.21\text{,}$$

(5.4) $$\tau^{32}=1.75\times 10^{-4}\sqrt{S_{12}^{2}}+1.50\text{,}$$

(5.5) $$\tau^{33}=5.31\times 10^{-4}\sqrt{S_{12}^{3}}+1.10\text{,}$$

where torsion tensors $S_{12}^{1}$, $S_{12}^{2}$ and $S_{12}^{3}$ correspond to dislocation tensor ${\tilde{\alpha }}^{31}$ (edge type), ${\tilde{\alpha }}^{32}$ (edge type) and ${\tilde{\alpha }}^{33}$ (screw type), respectively [50]. Therefore, equations (5.3), (5.4) and (5.5) are approximately the square-root notations of equation (4.6), which can express the type of dislocation (the orientation) in addition to the magnitude of the dislocation density [45].

The invariant of ${\tilde{\alpha }}^{\kappa \lambda }$ can be used as the scalar measure of density of GN dislocations and is defined as follows [51]:


(5.6) $$\begin{array}{lcr} & {\tilde{\alpha }}_{m}=\sqrt{\frac{2}{3}{\tilde{\alpha }}^{\kappa \lambda }{\tilde{\alpha }}^{\kappa \lambda }}. & \end{array}$$

**Figure 1.**
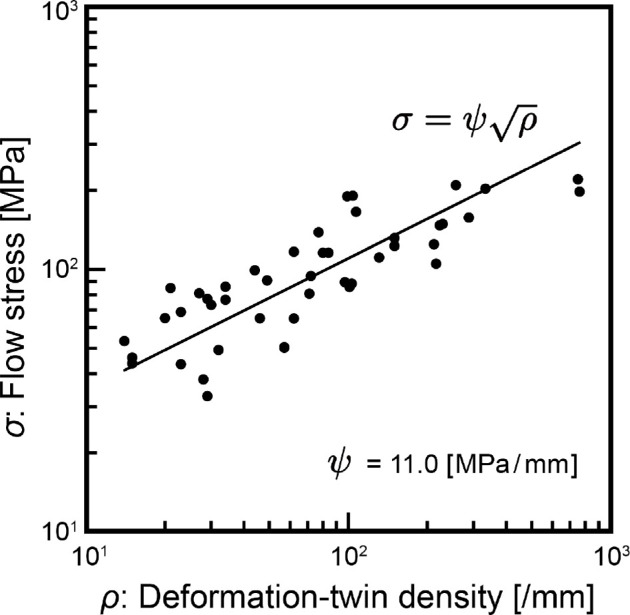
Relationship between flow stress (shear stress) and deformation-twin density of calcite. Data are from ST sections of Carrara marble in [6]. Results from SR sections are excluded due to high strain, which is probably influenced by other deformation mechanisms in addition to deformation twinning. SR sections are perpendicular to the sample axis, and ST sections are parallel to the sample axis. Results for Kunzendorfer marble and Solnhofen limestone are also excluded to eliminate the influence of grain size. See [6] for details.

Furthermore, ${\tilde{\alpha }}_{m}$ can be expressed as follows [51]:


(5.7) $$\begin{array}{lcr} & \rho_{g}=\frac{{\tilde{\alpha }}_{m}}{B}\text{,} & \end{array}$$

where $\rho_{g}$ is the density of GN dislocations and B is the magnitude of Burgers vector. Here, $B^{\kappa \lambda }$ in equations (2.1) and (2.2) is equivalent to the torsion tensor ${\tilde{\alpha }}^{\kappa \lambda }$, since the dislocation or Burgers vector can be represented by the torsion tensor as a topological singularity by a multi-valued displacement vector. When both ${\tilde{\alpha }}_{m}$ and $\rho_{g}$ in equation (5.7) are regarded as the scalar measure of deformation-twin density $B^{\kappa \lambda }$ and $\rho_{g}$ represents the dislocation density on deformation-twin boundary, $\rho_{g}$ can be equal to the deformation-twin density ρ in equation (1.1). Then, $\rho_{g}=\rho$ holds. Figure 1 shows the relationship between flow stress and deformation-twin density (number of deformation twins / [mm]) based on deformation experiments on calcite aggregates by Rybacki *et al*. [6]. The deformation-twin densities in figure 1 were observed in ST cross-sections parallel to the sample axis (see [6] for details). Although deformation-twin density is calculated using a one-dimensional linear method [52], it can be regarded as the deformation-twin boundary density from stereological perspectives and is a sufficient approximation of the true value when deformation-twin orientation is constrained [53]. In terms of equation (4.6), the relationship between deformation-twin density and flow stress (1.1) is well explained. Here, ρ in equation (1.1) represents the deformation-twin density (number of deformation twins / [mm]), which relates to the matrix width between twins $\overset{ˊ}{d}$ (i.e. twin spacing [mm]), as $\overset{ˊ}{d}\propto (\rho +1{)}^{-1}$ [53]. By replacing ρ in equation (1.1) with $\overset{ˊ}{d}$, equation (1.1) can be converted to equation (1.2). Thus, the relationship between flow stress and deformation-twin density (1.1) in Rybacki *et al*. [6] is equivalent to the Hall–Petch relationship, and strain hardening is due to increased deformation twins (i.e. decreased twin spacing). This supports the relationship between flow stress and deformation-twin density ρ in equation (1.1), interpreted as the dislocation density tensor $B^{\kappa \lambda }$ for deformation twins ($B^{\kappa \lambda }$ corresponding to ${\tilde{\alpha }}^{\kappa \lambda }$ in equation (4.6)), and reinforces the theoretical arguments in this study.

Similar relationships have been reported for deformation twins in metal and alloy [13,14]. Hence, the hardening caused by the formation of deformation twins is equivalent to the Hall–Petch relationship. The Hall–Petch relationship has also been observed in martensite block widths [54,55] and kink-band thicknesses [26]. This indicates that hardening results from the interfaces acting as barriers to dislocation movement. The Hall–Petch relationship has generally been proposed for the grain size (or grain boundaries). Deformation twins, as interfaces, were not counted as grains because deformation-twin boundaries are not considered strong against dislocation pile-ups [56]. However, the Hall–Petch effect has also been reported for deformation twins, kink bands and martensite, indicating that these crystal interfaces play a significant role in the Hall–Petch relationship and should not be neglected [4,54]. Rybacki *et al*. [4] proposed a model in which both grain boundaries and deformation-twin boundaries affect the movement of free dislocations in calcite. As an example, transmission electron microscopy (TEM) observations show increased dislocation density near the deformation-twin boundaries [6]. Frequently, the matrix dislocations are arranged in cell-like networks suggesting repulsive interactions between deformation twins and the dislocation structure [6]. It is also pointed out that the annealing twin boundary should be treated similarly to grain boundary in terms of the Hall–Petch relationship [57,58]. Moreover, it has been reported that the influence of interfaces on dislocation movement depends on crystal structure, particularly slip systems [5,59]. Therefore, further research is expected to establish the Hall–Petch relationship that incorporates the effects of both grain boundary and interfaces such as deformation-twin boundary.

In contrast, interfaces affect the movement of dislocations and cause hardening, but at the nanoscale (nanograin, nanotwinned Cu and nanotwinned Au nanowires) softening is reported despite the large number of interfaces due to the inverse Hall–Petch relationship [18,19,60]. This is because the strain is accumulated by grain boundary sliding rather than dislocation slip in grains due to the smaller grain size [18,61]. This indicates that the interaction between the deformation-twin boundary and grain boundary can also induce softening. The volume fraction of triple junctions or quadruple junctions for grain boundaries is an important factor when considered in three dimensions at the nanoscale [62–64]. The higher volume fractions of triple junctions or quadruple junctions can be attributed to the fact that the strength of the interface is no longer supported by the rank-1 connection (refer to appendix A for details). This suggests that the inverse Hall–Petch relationship holds at the nanoscale level.

In polycrystalline materials, deformation twinning and grain boundary plasticity are strongly coupled during deformation, dynamically changing the configuration of hierarchical twin boundary-grain boundary network, and dense networks of deformation twins endow metals and alloys with unprecedented mechanical properties [65]. For example, by creating a hierarchical structure of twinned nanograins having the size in the Hall–Petch region but close to the inverse Hall–Petch region, a high yield strength was obtained [66]. Therefore, from the viewpoint of the rank-1 connection, further research on hierarchical structures of twinned nanograins is needed.

Finally, we discuss the equivalence among different interface theories. As previously mentioned, the surface dislocation theory (§2) [21] is equivalent to the geometrical conditions of compatibility of the first order for discontinuity (3.9) and the Hadamard jump condition. These interface theories are also equivalent to rank-1 connection and 0-lattice theory [31]. The details of rank-1 connection [67] and 0-lattice theory are summarized in appendix A. It should be noted that while surface dislocation theory and geometrical conditions of compatibility of the first order for discontinuity are used in differential geometry, making them applicable to curved surfaces, rank-1 connection and the 0-lattice theory are matrix-based formulations with different application limitations. Nevertheless, it is indicated that deformation-twin, martensite and kink boundaries cause strain hardening and satisfy the rank-1 connection [67,68].

## 6. Conclusion

We propose that the Bailey–Hirsch relationship between the deformation-twin density and flow stress can be expressed in tensor notation within the framework of surface dislocation theory. This relationship indicates that an increase in the deformation-twin density (i.e. an increase in the deformation-twin density tensor) causes hardening. By replacing the deformation-twin density with the deformation-twin spacing, the Hall–Petch relationship for the deformation-twin spacing can be derived. This relationship is consistent with the results from deformation experiments on calcite aggregates. The inverse Hall–Petch relationship for a nanosized deformation twin or grain size is explained from the viewpoint that nanosized grain size cannot satisfy the rank-1 connection. Finally, we demonstrated the mathematical equivalence among interface theories including surface dislocation theory, the geometrical conditions of compatibility of the first order for discontinuity, Hadamard jump condition, rank-1 connection and 0-lattice theory. Thus, interfaces such as deformation-twin, kink and martensite boundaries, which satisfy the rank-1 connection, can be comprehensively understood to cause strain hardening.

## Figures and Tables

**Figure 2.**
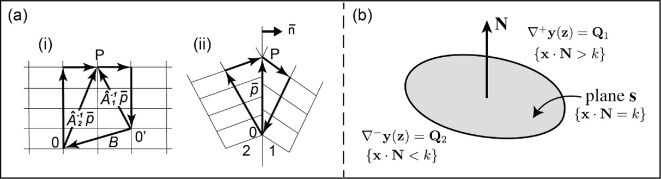
(a) Figures to supplement (A 1) in the orthogonal Cartesian coordinate system modified from the work of Hashimoto [70]. (i) Reference lattice with origin 0. The Burgers vector B is present in the Burgers circuit 00'P. (ii) Lattices 1 and 2 are created from the reference lattice in (i) via affine transformations ${\hat{A}}_{1}$ and ${\hat{A}}_{2}$. (b) Correspondence of the Hadamard jump condition and rank-1 connection in the orthogonal Cartesian coordinate system modified from the work of Ball [71].

**Figure 3.**
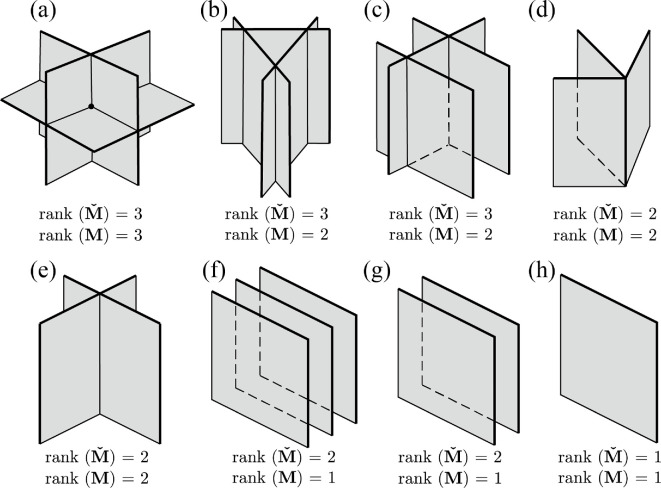
Solutions of simultaneous linear equation (B 1) and arrangement of planes modified after Anton [78]. (a) One solution (three planes intersecting at a point). (b) No solutions (three planes with no common intersections). (c) No solutions (two parallel planes intersecting a third plane). (d) Infinitely many solutions (three planes intersecting in a line). (e) Infinitely many solutions (two coincident planes intersecting a third plane). (f) No solutions (three parallel planes). (g) No solutions (two coincident planes parallel to a third plane). (h) Infinitely many solutions (three coincident planes).

## Data Availability

Data relating to figure 1 are available in the electronic supplementary material [69].

## Appendix A. Mathematical correspondence among surface dislocation theory, rank-1 connection, Hadamard jump condition and 0-lattice theory

The significance of the rank-1 connection in materials science and the mathematical correspondence among the rank‐1 connection, the surface dislocation theory, and the 0-lattice theory are briefly introduced.

First, we explain surface dislocation theory using figure 2a, citing Hashimoto [70]. Consider the situation in figure 2a(i),(ii) after deformation. ${\hat{A}}_{1}$ and ${\hat{A}}_{2}$ denote the transformations (3 × 3 matrices) that create lattices 1 and 2 during deformation. The two lattices meet along an interface with a unit normal $\bar{n}$ and vector $\bar{p}$ in the plane. If lattice 1 is selected as the reference lattice and the transformation from lattice 1 to lattice 2 is denoted by $\hat{A}$, the Burgers vector B is expressed as follows:

(A 1)
$$\begin{array}{lcr} & B=\left(I-{\hat{A}}^{-1}\right)\bar{p}\text{,} & \end{array}$$

where I denotes an identity matrix. Here, we review the theoretical equations that describe the geometric connection conditions of the interface, including equation (A 1), and their correspondence.

Next, the rank-1 connection is explained, citing Ball [71,72] and Šilhavý [73]. Consider a smooth surface s containing a point $z\in {\mathbb{R}}^{3}$ with unit normal N to s (figure 2b). We have $\nabla^{+}\text{y}(\text{z})={\text{Q}}_{1}$ and $\nabla^{-}\text{y}(\text{z})={\text{Q}}_{2}$ from above and below s{x⋅N=k}, respectively. Let $\text{Q}={\text{Q}}_{1}-{\text{Q}}_{2}$. Then, Qx=0 if x⋅N=0. Thus, Q(x−(x⋅N)N)=0 for all z, and Qz=(QN⊗N)z. Therefore, the following Hadamard jump condition is derived:

(A 2)
$$\begin{array}{lcr} & {\text{Q}}_{1}-{\text{Q}}_{2}=\text{a}⊗\text{N}\text{.} & \end{array}$$

For a vector $\text{a}\in {\mathbb{R}}^{3}$, the right-hand side of equation (A 2) is a 3×3 matrix of rank 1 (provided a≠0) with entries $(\text{a}⊗\text{N}{)}_{ij}=a_{i}N_{j}$. Here, considering **a** as a vector representing the discontinuity of the deformation gradient, it becomes a condition for maintaining the continuity of the deformation at the interface s when deformation gradients ${\text{Q}}_{1}$ and ${\text{Q}}_{2}$ are applied, and equation (A 2) is equivalent to the rank-1 connection [29]. Thus, the Hadamard jump condition is rank-1 of a matrix in linear algebra (see appendix B) and can be regarded as a condition for a rank-1 connection [29,74]. Considering the relationship between the rank of simultaneous linear equations and solutions from the perspective of material science, the rank-1 connection is a condition in which both sides are connected at the interface. In other words, a rank-1 connection is a geometric condition necessary and sufficient for connecting two different areas of uniform deformation [29] (see appendix B).

Moreover, we briefly review 0-lattice theory with reference to the work of Bollmann [31,75,76]. When the crystal lattices on both sides are virtually extended and superimposed, a transformation operation ($\bar{A}$) is considered that causes the crystal lattice points on both sides to have a one-to-one correspondence projection. Points that can be the origin of the conversion operation are regularly arranged, forming a lattice of their own. This collection of origins is defined as the 0-lattice. When lattice 2 is created from lattice 1 via affine transformation, if any point $x^{\left(1\right)}$ of lattice 1 is equivalent to $x^{\left(2\right)}$ after the transformation, the following relationship holds for the affine transformation matrix $\bar{A}$:

(A 3)
$$\begin{array}{lcr} & x^{\left(2\right)}=\bar{A}x^{\left(1\right)}\text{,} & \end{array}$$

where $x^{\left(2\right)}$ is the 0-point. The following is obtained using the translation vector $t^{\left(1\right)}$ of lattice 1:

(A 4)
$$\begin{array}{lcr} & x^{\left(2\right)}=x^{\left(1\right)}+t^{\left(1\right)}\equiv x^{\left(0\right)}. & \end{array}$$

Therefore, from equations (A 3) and (A 4), we[Disp-formula A1-E3] obtain

(A 5)
$$\begin{array}{lcr} & x^{\left(0\right)}={\bar{A}}^{-1}x^{\left(0\right)}+t^{\left(1\right)}. & \end{array}$$

The following equation holds

(A 6)
$$\begin{array}{lcr} & \left(I-{\bar{A}}^{-1}\right)x^{\left(0\right)}=B^{\left(L\right)}\text{,} & \end{array}$$

where I represents the unit transformation matrix and $B^{\left(L\right)}$ is an arbitrary translation vector of lattice 1. Here, equation (A 6) is the basic equation of the 0-lattice theory and is also a generalized equation of Frank’s formula [77] for low-angle grain boundaries [76].

In this appendix, the correspondence among the rank-1 connection, 0-lattice theory, and surface dislocation theory is explained according to the notation of original papers. 0-lattice theory equations are satisfied when the lattice is joined in the planes before and after deformation. Here, 0-lattice theory (A 6) is mathematically equivalent to surface dislocation theory (A 1). Moreover, equation (A 6) is mathematically equivalent to equation (A 2), and lattices 1 and 2 are connected on the plane when the ranks of the matrices $\left(I-{\bar{A}}^{-1}\right)$ of equation (A 6) and $\left(I-{\hat{A}}^{-1}\right)$ of equation (A 1) are 1. In other words, the condition of rank-1 connection is satisfied. In addition, surface dislocation theory can be applied to diverse curved matter spaces in continuum mechanics without special crystal orientation, but not in 0-lattice theory.

## Appendix B. Geometric meaning of matrix rank

In this appendix, the geometric meaning of a rank-1 connection is explained from the perspective of linear algebra. In the three-dimensional Euclidean space, the simultaneous linear equations for the three variables X, Y and Z are as follows:

(B 1)
$$\begin{array}{lcr} & \left\{ \;\begin{array}{rl} & aX+bY+cZ=K_{1}\\ & dX+eY+fZ=K_{2}\\ & gX+hY+iZ=K_{3} \end{array}.\right. & \end{array}$$

Equation (B 1) is summarized according to the definition of the matrix product as follows:

(B 2)
$$\begin{array}{lcr} & \text{M}\text{X}=\text{K}\text{,} & \end{array}$$

where

(B 3)
$$\begin{array}{lcr} & \text{M}=\left[\begin{array}{lll} a & b & c\\ d & e & f\\ g & h & i \end{array}\right]\text{,}\;\text{X}=\left[\begin{array}{l} X\\ Y\\ Z \end{array}\right]\text{,}\;\text{K}=\left[\begin{array}{l} K_{1}\\ K_{2}\\ K_{3} \end{array}\right]. & \end{array}$$

The matrix **M** on the left side of equation (B 2) is a coefficient matrix. The augmented coefficient matrix in (B 1) is expressed as follows:

(B 4)
$$\begin{array}{lcr} & \overset{ˇ}{\text{M}}=\left[\begin{array}{cc} \text{M} & \text{K} \end{array}\right]=\left[\begin{array}{cccc} a & b & c & K_{1}\\ d & e & f & K_{2}\\ g & h & i & K_{3} \end{array}\right]. & \end{array}$$

Equation (B 1) has either (i) one solution, (ii) no solutions, or (iii) infinitely many solutions, and it expresses the relationship between the coefficient matrix **M** and augmented coefficient matrix $\overset{ˇ}{M}$ in equation (B 4), as shown in table 1. Figure 3 illustrates the geometric possibilities that occur when equation (B 1) has zero, one, or infinite solutions. When rank ($\overset{ˇ}{M}$) = rank (**M**) = 1, the solutions exist in one plane, as shown in figure 3h. In other words, ‘rank-1’ of rank-1 connection implies that the two sides are connected as planes.

**Table 1.** Relationships between the number of solutions to equation (B 1) and rank ($\overset{ˇ}{M}$) and rank (**M**).

rank ($\overset{ˇ}{M}$)	rank (M)	number of solutions
3	3	one solution (on a point, i.e. 0 degrees of freedom)
3	2	no solutions
2	2	infinitely many solutions (on a line, i.e. 1 degree of freedom)
2	1	no solutions
1	1	infinitely many solutions (on a plane, i.e. 2 degrees of freedom)

The rank-1 connection condition applies the degrees of freedom of the solutions (=n−r, where n represents the dimension of the matrix and r represents the rank of the matrix) to the simultaneous linear equations of linear algebra to the fit condition of the deformation. Therefore, it is suggested that when the rank of the solution to a simultaneous linear equation is 1, a rank-1 connection is satisfied and strain hardening occurs.
